# Performance of the automated Sysmex XN-3000 analyser for detecting white blood cell abnormalities in South Africa

**DOI:** 10.4102/ajlm.v12i1.2140

**Published:** 2023-07-19

**Authors:** Jasmine Ramiah, Dashini Pillay, Nadine Rapiti

**Affiliations:** 1 Department of Haematology, National Health Laboratory Services, Inkosi Albert Luthuli Central Hospital, Durban, South Africa; 2 Department of Haematology, School of Laboratory Medicine, University of KwaZulu-Natal, Durban, South Africa

**Keywords:** Sysmex analyser, XN-series, automated analysers, FBC, flagging, white blood cell flags, Clinical and Laboratory Standards Institute guidelines

## Abstract

**Background:**

Automated haematology analysers such as the Sysmex XN-3000 (Sysmex Corporation, Kobe, Japan) utilise white blood cell (WBC) flags to identify quantitative and qualitative abnormalities. Owing to clinical and biological factors, the sensitivity and specificity of the flags vary when compared to microscopy, the gold-standard method for assessing peripheral blood smear (PBS) morphology.

**Objective:**

This study assessed the performance of the Sysmex XN-3000 haematology analyser in comparison to PBS microscopy for the detection of WBC abnormalities.

**Methods:**

We collected 250 random full blood count samples from the haematology laboratory at Inkosi Albert Luthuli Central Hospital, Durban, KwaZulu-Natal, South Africa, from March 2022 to April 2022. The performance of the automated WBC flags of the Sysmex XN-3000 was assessed in comparison to PBS microscopy, and the impact of established clinical variables on the performance of the flags was determined.

**Results:**

The sensitivity of the ‘blast’ flag was 96.3%, and the specificity was 84.9%. The efficiency of the flag was adversely impacted by low white cell counts (< 1.5 × 10^9^/L; *p* < 0.001), chemotherapy (*p* = 0.002), malignancy (*p* = 0.02), and infection (*p* = 0.02). The ‘abnormal lymphocyte’ flag demonstrated a sensitivity of 90% and a specificity of 96.2%, and its performance was adversely impacted by chemotherapy exposure (*p* = 0.03). Three cases (1.2%) erroneously flagged as ‘monocytosis’ demonstrated blasts on microscopy.

**Conclusion:**

In our setting, PBS microscopy remains necessary to confirm blasts, abnormal lymphocytes, and monocytosis in patients with malignancy, current chemotherapy exposure, low white cell counts, and infection.

**What this study adds:**

This study adds evidence that PBS morphology remains the gold standard for confirming WBC abnormalities in patients with a history of malignancy, chemotherapy, and leucopenia.

## Introduction

The microscopic assessment of a well-prepared and well-stained peripheral blood smear (PBS) is the gold standard for identifying white blood cell (WBC) abnormalities.^1^ This manual PBS assessment requires skilled and trained morphologists.^2^

Automated full blood count (FBC) haematology analysers can flag suspected quantitative and qualitative abnormalities,^1^ and the WBC flags can potentially alert the laboratory technical staff to abnormal blood samples prior to the review of the PBS, thus improving workflow, particularly in resource-constrained settings. Globally, restricted laboratory budgets result in a reduction in the number of experienced morphologists.^3^ A laboratory system with automated technology that can prioritise a blood sample with abnormal WBC features is thus advantageous. However, there are limitations in the specificity and sensitivity of these flags.^4^ The Sysmex XN-3000 automated FBC analyser (Sysmex Corporation, Kobe, Japan) has white cell differential, white cell nucleated, and white cell precursor (WPC) channels, with the WPC channel demonstrated to possess an improved sensitivity for detecting blasts.^5^

The National Health Laboratory Services haematology laboratory at Inkosi Albert Luthuli Central Hospital, located in Durban, KwaZulu-Natal, South Africa, processes approximately 9000 FBC samples and 300–600 PBS samples monthly. The Inkosi Albert Luthuli Central Hospital is a referral hospital that provides medical care to patients with benign and malignant haematological conditions.^6^

The purpose of this study was to assess the performance of the WBC flags of the Sysmex XN-3000 analyser (Sysmex Corporation, Kobe, Japan) for the detection of WBC abnormalities in comparison to PBS microscopy, which is used as the gold-standard method in our setting. We also aimed to identify the clinical variables in our hospital setting that may be associated with false-positive (FP) and false-negative (FN) WBC flags.^2^

## Methods

### Ethical considerations

The study was approved by the University of KwaZulu-Natal Biomedical Research Ethics Committee (ethical clearance number: BREC/00003769/2022). Informed consent was not required by the Ethics approval committee as these samples were routinely performed by the clinician. The clinician had obtained consent for all laboratory procedures prior to admission of the patients and the laboratory had waived need for consent as no additional tests were performed. Full BREC ethics approval was obtained for this study. Informed consent was not obtained as these samples were submitted to the laboratory for routine FBC analysis and PBS microscopy by the treating clinician. Patient confidentiality was maintained during the study by the allocation of unique and anonymised study codes. The research data were stored electronically on password-protected devices accessible only by the researchers.

### Study design

This was a laboratory-based prospective study conducted in the National Health Laboratory Services, Durban, KwaZulu-Natal, South Africa. The study comprised 250 blood samples, which were representative of the normal and abnormal adult patient population (> 12 years of age) sent to the laboratory for routine FBC analysis in dipotassium ethylene diamine tetra-acetate tubes (Becton Dickinson, Berlin, Germany).

The sample size was calculated using Stata^®^ V15.1 statistical software (StataCorp LLC, College Station, Texas, United States) in consultation with a statistician who audited the number of monthly FBC requests. The samples were collected from March 2022 to April 2022.

The results were quality assured by assessing the instrument’s daily internal quality controls and external quality assurance, according to the department’s standard operating procedure.^6^

### Automated white blood cell analysis

The anticoagulated samples were drawn and analysed within 24 h of sampling on the Sysmex XN-3000 analyser (Sysmex Corporation, Kobe, Japan) for FBC and WBC differential count analysis. If the sample demonstrated ‘blast’, ‘atypical lymphocytes’, or ‘abnormal lymphocytes’, the WPC channel was reflexed and run according to the routine procedure.^5^ Samples that were clotted or haemolysed, had insufficient volume, were older than 24 h, or were collected in an incorrect tube or erroneously labelled were excluded from the study. The Sysmex XN-3000 analyser uses the principles of fluorescent flow cytometry to identify WBC sub-populations based on the intensity of the side-fluorescent light and side-scattered light.^5,7^ The initial reflex or warning is identified by the white cell differential, which generates an internal protocol message or flag. This flag is further confirmed or removed by the more sensitive WPC channel.^5,8^

### Peripheral blood smear analysis

A PBS was prepared from each blood sample and stained according to the May-Grunwald Giemsa technique using the automated slide stainer (Sysmex P10 slide Maker and Stainer, Sysmex Corporation, Kobe, Japan) according to the Clinical and Laboratory Standards Institute recommendations.^9^ Each PBS was microscopically assessed (Olympus CX23, Evident Corporation, Tokyo, Japan) at 10× and 50× magnification with oil by the principal investigator and a trained haematology laboratory technologist. A 100 WBC differential count was performed for each sample. The machine differential and manual differential counts were entered into Microsoft^®^ Excel 2016 spreadsheets (Microsoft^®^, Redmond, Washington, United States).

### Clinical information

We collected the following clinical information from the hospital and laboratory information systems: age, sex, white cell count (WCC), presence of an infection, coronavirus disease 2019 active infection or previous coronavirus disease 2019 infection, clinical diagnosis, transplant status, current chemotherapy exposure, and HIV status. Infection, as recorded in the clinical information, was determined by a temperature of ≥ 38 °C or culture of an organism (bacterial, viral, etc.) from any clinical site, or a clinical site of infection.^10^

The reference range for WCC in our setting ranges from 3.92 to 10.40 × 10^9^/L for male patients and from 3.90 to 12.60 × 10^9^/L for female patients. In our study, a low WCC was defined as a count less than 1.5 × 10^9^/L.^6^

### Data analysis

Statistical data were captured using Microsoft^®^ Excel 2016 (Microsoft^®^, Redmond, Washington, United States) and analysed using R Statistical Computing Software of the R Core Team, 2020, version 3.63 (Microsoft, Windows [32/64 bit], Redmond, Washington, United States). All the numeric variables were skewed, and the descriptive statistics were consequently presented as medians with lower and upper quartiles. The categorical variables were presented as counts and percentage frequencies. The association between the numeric variables was determined using Pearson’s correlation test. All the statistical tests were conducted at a 5% level of significance. A *p*-value of < 0.05 was considered significant for this study.

Sensitivity and specificity analyses were calculated using the ‘epi.tests’ R function from the epiR package (https://cran.r-project.org/package=epiR) while the McNemar test was performed using the epi.kappa function also from the epiR package. A ‘true-positive’ (TP) was defined as a WBC abnormality detected by the analyser and confirmed by PBS microscopy. A ‘false positive’ (FP) was defined as a WBC abnormality detected by the analyser but not observed on PBS microscopy. A ‘true-negative’ (TN) was defined as a WBC abnormality not detected by the analyser and not observed on PBS microscopy. A ‘false negative’ (FN) was defined as a WBC abnormality not detected by the analyser but observed on PBS microscopy. The equations used for the sensitivity, specificity, positive predictive value (PPV), and negative predictive value (NPV) calculations using the epiR package are shown below:

sensitivity=TP/(TP+FN) [Eqn 1]

specificity=TN/(TN+FP) [Eqn 2]

positive predictive value (PPV)=TP/(TP+FP) [Eqn 3]

negative predictive value (NPV)=TN/(FN+TN) [Eqn 4]

The impact of the clinical variables (presence of infection, malignancy, current chemotherapy usage and low WCC [< 1.5 × 10^9^/L]) on the WBC flags was assessed using the McNemar test when the WBC flag did not correlate (FP or FN) with the microscopy results.

## Results

### Study population

This study included 250 adult patient samples (Table 1). One hundred and thirteen (45.2%) of the patients were men and 137 (54.8%) were women. One hundred and forty-five samples (56%) were from the haematology or oncology clinic or ward. Of the total 250 study samples, there were 125 normal (no quantitative or qualitative WBC abnormalities present) and 125 abnormal (flagged by the analyser as having WBC abnormalities) samples.

**TABLE 1:** Demographic characteristics and clinical information of the study population, Inkosi Albert Luthuli Central Hospital, Durban, KwaZulu-Natal, South Africa, March 2022 – April 2022.

Characteristic (*n* = 250)	*n*	%
**Gender**		
Male	113	45.2
Female	137	54.8
**White cell count range** †		
White cell count < 1.5 × 10^9^/L	37	14.8
White cell count 1.5 × 10^9^/L – 12 × 10^9^/L	169	67.6
White cell count > 12 × 10^9^/L	44	17.6
**Age range**		
≤ 30 years	100	40.0
31–59 years	121	48.4
≥ 60 years	29	11.6
**COVID-19**		
No previous or current COVID-19 infection	223	89.2
Current COVID-19 infection	7	2.8
Previous COVID-19 infection	20	8.0
**Disease entities**		
Haematological malignancy	112	44.8
Non-haematological malignancy	50	20.0
Benign haematological disorders	22	8.8
Dermatological	15	6.0
Autoimmune disorders	12	4.8
Inflammatory diseases	11	4.4
Post-haematopoietic stem cell transplant	8	3.2
HIV-positive	28	11.2
Chemotherapy	103	41.2
Infection	74	29.6
Miscellaneous disorders‡	29	11.6

COVID-19, coronavirus disease 2019.

† , The normal white cell count reference range in our setting is 3.92 – 10.40 × 10^9^/L for men and 3.90 – 12.60 × 10^9^/L for women;

‡ , Miscellaneous disorders include trauma, stem cell donors, post-solid organ transplantation, cardiovascular disease, renal disease, genetic disorders, infection, endocrinopathy, respiratory disease and thrombophilia.

### Clinical characteristics

Of the 112 (44.8%) patients with a haematological malignancy, 51 (20.4%) had acute myeloid leukaemia, 36 (14.4%) had acute lymphoblastic leukaemia or lymphoma, 10 (4%) had a lymphoproliferative disorder, and 8 (3.2%) had a plasma cell dyscrasia. One hundred and three (41.2%) patients were on chemotherapy, 74 (29.6%) had an infection, and 8 (3.2%) patients had received a haematopoietic stem cell transplant.

Twenty-two (8.8%) samples were from patients with benign haematological disorders such as thalassaemia major, aplastic anaemia and sickle cell disease. Among the 28 (11.2%) HIV-positive patients, the CD4 count ranged from 35 × 10^9^/L to 1233 × 10^9^/L, and viral load ranged from 0 copies/mL to 656 000 copies/mL.

### White cell count

The WCC among the study subjects ranged from 0.1 × 10^9^/L to 145.5 × 10^9^/L (median: 5.24 × 10^9^/L; interquartile range: 2.43–8.67 × 10^9^/L). Thirty-seven samples (14.8%) demonstrated a WCC ≤ 1.5 × 10^9^/L, 169 (67.6%) demonstrated a WCC between 1.6–11.9 × 10^9^/L, and 44 samples (17.6%) demonstrated a WCC of ≥ 12 × 10^9^/L. The blast percentage ranged from 0% to 78%.

There were agreements (*p* < 0.001) between the PBS microscopy and the numerical WBC values noted on the Sysmex XN-3000 analyser for the median differential counts of neutrophils (54 × 10^9^/L vs 55.4 × 10^9^/L), lymphocytes (26 × 10^9^/L vs 29.1 × 10^9^/L), immature granulocytes (0 × 10^9^/L vs 0.50 × 10^9^/L), and monocytes (7 × 10^9^/L vs 7.55 × 10^9^/L) (Table 2). The basophils, however, did not demonstrate an agreement as the median count was 0 × 10^9^/L by PBS and 0.40 × 10^9^/L by the automated analyser (*p* = 0.494).

**TABLE 2:** Correlation between white blood cell differential count by the Sysmex XN-3000 analyser and white blood cell differential count by microscopy, Inkosi Albert Luthuli Central Hospital, Durban, KwaZulu-Natal, South Africa, March 2022 – April 2022.

White blood cell flag	Median microscopy differential count (× 10^9^/L)	Median Sysmex XN-3000 differential count (× 10^9^/L)	Correlation between the Sysmex XN-3000 analyser and microscopy†	*p*
Neutrophils	54	55.4	0.890	< 0.001
Lymphocytes	26	29.1	0.766	< 0.001
Immature granulocytes	0	0.50	0.562	< 0.001
Monocytes	7	7.55	0.268	< 0.001
Basophils	0	0.40	−0.043	0.494

† , Pearson’s correlation was applied to determine the association between the white blood cell differential counts obtained using microscopy and the automated analyser.

Sixty-two (24.8%) samples were flagged for ‘blast’ by the analyser, 47 (18.8%) were flagged for ‘immature granulocytes’, and 43 (17.2%) were flagged for ‘abnormal lymphocytes’.

The efficiencies of the ‘conspicuous eosinophil’, ‘lymphocytosis’, ‘atypical lymphocyte’, and ‘immature granulocyte’ flags were not impacted by the clinical parameters (*p* > 0.05) (Table 3).

**TABLE 3:** Performance of the Sysmex XN 3000 Analyser for the detection of white blood cell abnormalities and the impact of clinical variables on the performance of WBC flags, Inkosi Albert Luthuli Central Hospital, Durban, KwaZulu-Natal, South Africa, March 2022 – April 2022.

White cell flag	Sensitivity (%)	Specificity (%)	PPV (%)	NPV (%)	Low white cell count (≤ 1.5 × 10^9^/L) (*p*)	Chemotherapy† (*p*)	Malignancy (*p*)	Infection (*p*)
Conspicuous eosinophil	50.0	100.0	50.0	100.0	0.9	0.4	0.9	0.9
Atypical lymphocyte	26.1	99.5	92.3	85.7	0.2	0.7	0.6	0.1
Abnormal lymphocyte	90.0	96.2	81.8	98.1	0.4	0.03	0.2	0.3
Lymphocytosis	97.8	100.0	100.0	99.5	0.7	0.4	0.2	0.5
Left shift	59.7	95.1	78.7	95.1	0.2	0.3	0.005	0.44
Immature granulocyte	78.7	98.9	95.6	85.9	0.5	0.9	0.36	0.12
Blast	96.3	84.9	44.1	99.5	< 0.001	0.002	0.02	0.02
Monocytosis	84.3	97.0	69.6	98.7	0.2	0.4	0.08	0.6

Note: The PPV and NPV were calculated using sensitivity and specificity analysis or decision theory.

PPV, positive predictive value; NPV, negative predictive value.

† , The McNemar test was used to determine the effect of the clinical parameters on the performance of the automated analyser.

The ‘abnormal lymphocyte’ WBC flag generated by the instrument demonstrated a sensitivity of 90% (95% confidence interval [CI]: 76.3% – 97.2%), a specificity of 96.2% (95% CI: 92.6% – 98.3%), a PPV of 81.8%, and an NPV of 98.1%. On comparing the analyser results to microscopy, four samples (1.6%) that were not flagged for ‘abnormal lymphocytes’ by the analyser were determined to be FN, and eight (3.2%) samples were FP for ‘abnormal lymphocytes’.

The ‘blast’ flag generated by the instrument demonstrated a sensitivity of 96.3% (95% CI: 81% – 99.9%), a specificity of 84.9% (95% CI: 79.5% – 89.4%), a PPV of 44.1%, and an NPV of 99.5%. One (0.4%) sample was FN for ‘blast’, and 33 (13.2%) were FP. The blast count ranged from 0% to 78%. The accuracy of the ‘blast’ flag was influenced by a low WCC (*p* < 0.001), the presence of malignancy (*p* = 0.002), current chemotherapy exposure (*p* = 0.02), and infection (*p* = 0.02).

The monocytosis ‘flag’ generated by the instrument demonstrated a sensitivity of 84.3% (95% CI: 60.4% – 96.6%), a specificity of 97.0% (95% CI: 93.9% – 98.8%), a PPV of 69.6%, and an NPV of 98.7%. Seven (2.8%) samples flagged for monocytosis by the automated analyser were determined to be FP for monocytosis and three (1.2%) samples not flagged by the analyser were determined to be FN. These three (1.2%) samples had > 20% blasts on manual microscopic assessment of the PBS.

The ‘left shift’ flag generated by the instrument demonstrated a sensitivity of 59.7% (95% CI: 47.5% – 71.1%), specificity of 95.1% (95% CI: 96% – 99.9%), a PPV of 95.6%, and an NPV of 85.9%. Twenty-nine (11.6%) samples were determined to be FN and two (0.8%) samples were FP. The accuracy of this flag was influenced by current chemotherapy exposure (*p* = 0.005).

## Discussion

We evaluated the performance of the Sysmex XN-3000 automated analyser for the detection of WBC abnormalities in 250 routine adult blood samples at the National Health Laboratory Services laboratory at Inkosi Albert Luthuli Central Hospital, Durban, KwaZulu-Natal, South Africa. The majority of the samples were from patients diagnosed with a malignancy and patients receiving chemotherapy, which was expected as this is a tertiary referral centre for haematological and oncological disorders. Congruent with reports in the literature, the accuracy of the ‘blast’, ‘abnormal lymphocytes’, ‘left shift’ and ‘monocytosis’ automated WBC flags were impacted by clinical parameters such as current infection, low WCC (≤ 1.5 × 10^9^/L), malignancy, and current treatment with chemotherapy.^11^ Failure to identify malignant conditions portends significant clinical implications and is one of the greatest concerns for laboratories. Therefore, in these instances, laboratories should rely on the confirmatory result of a detailed microscopic assessment, which is the gold standard.^12^

There was an agreement between the Sysmex XN-3000 analyser and PBS microscopy for the differential counts of neutrophils, lymphocytes, immature granulocytes, and monocytes (*p* < 0.001). However, there was no agreement between the Sysmex XN-3000 analyser and PBS microscopy for the differential count of basophils. The automated analyser does not quantify the presence of blasts and abnormal or atypical lymphocytes; it only provides a qualitative assessment through the presence of flags. Therefore, a quantitative agreement between microscopy and the analyser could not be determined.

The ‘blast’ flag indicates the suspected presence of malignancy (myeloid or lymphoid).^13^ Although the WPC, employed by the XN-series, has improved the detection of blasts, the literature demonstrates a low specificity and variable sensitivity (60% – 100%).^4^ A study conducted in Denmark in 2020 revealed that the WPC produced a high number of FP (14.5%) and FN (12.8%) results when detecting blasts, possibly due to leucopenia and a low number of circulating blast cells.^4^ Our study demonstrated a sensitivity of 96.3% and a specificity of 84.9% for the blast flag, and the blast count ranged from 0% to 78%. The sensitivity and specificity of the blast WBC flag were determined to be influenced by leucopenia (WCC of ≤ 1.5 × 10^9^/L). The lower the WCC, the higher the likelihood of erroneous blast detection by the analyser. This is similar to a previous report in Korea in 2021 where the sensitivity increased as the WBC rose to > 6 × 10^9^/L.^2^ Although in our study, the automated analyser did not detect the presence of blasts in one sample (0.4%) from a patient with leucopenia (WCC: < 1.5 × 10^9^/L), malignancy and current chemotherapy exposure, 21% blasts were detected by microscopy in that sample. In the Korean study, the sensitivity of the WPC in detecting blasts in leucopenic samples (< 1.5 × 10^9^/L) was 56%.^2^ This may be explained by patients receiving chemotherapy or transplantation, which may alter the side-fluorescent light.^2^ In the case of leucopenia, blasts may be further missed due to infrequency.^4^ Our study demonstrated that microscopy remains the gold standard in the detection of blasts in patients with a low WCC, patients with a malignancy, and patients receiving chemotherapy.

The ‘atypical lymphocyte’ flag indicates reactive disorders (infections or inflammatory states).^14^ The morphology may be heterogenous, including larger size, round nucleus, larger nucleolus, abundant deep basophilic cytoplasm with tenting, or the presence of intracytoplasmic granules.^15^ According to a local study conducted at Charlotte Maxeke Johannesburg Academic Hospital, South Africa, in 2018, the ‘atypical lymphocyte’ flag was associated with a high FP rate of 41.74%.^7^ Inflammatory states such as HIV may affect the sensitivity of the atypical lymphocyte and abnormal lymphocyte channel of the automated analyser.^7^ This, however, was not proven in our study possibly due to the low number of HIV-positive patients in the study (11.2%), which is lower than the 27% prevalence of HIV in KwaZulu-Natal.^16^ Schapkaitz and Khoza previously demonstrated the sensitivity and specificity of the ‘atypical lymphocyte’ flag in patients with HIV in the South African setting to be 100% and 74.67%.^7^ Our study revealed the reverse: a sensitivity of 26.1% and a specificity of 99.5%. Our study also demonstrated that the accuracy of the atypical lymphocyte flag was not significantly influenced by the clinical parameters.

‘Abnormal lymphocytes’ indicates a malignant lymphoid process which could be acute, chronic, indolent, or high grade.^17^ Distinguishing reactive lymphocytes secondary to a viral aetiology from those arising from a lymphoproliferative disorder is challenging due to morphological heterogeneity.^7^ According to a local study conducted in Charlotte Maxeke Johannesburg Academic Hospital, South Africa, in 2018, the FP rate for abnormal lymphocyte flags using the Sysmex XN-9000 was 55.56% due to the presence of HIV, opportunistic infections, and lymphopenia.^7^ The study also demonstrated a sensitivity of 88.46% and a specificity of 74.6% for the ‘abnormal lymphocyte’ flag. In our study, the FP rate was 3.2%, and we observed a higher sensitivity (90%) and specificity (96.2%). In our study, four (1.6%) samples confirmed to be positive for abnormal lymphocytes using PBS microscopy were FN for ‘abnormal lymphocytes’ on the analyser. The clinical parameters in those samples included recent chemotherapy exposure, malignancy, and infection; two samples were unexplained. Our study also demonstrated reduced reliability of the ‘abnormal lymphocyte’ flag in the presence of chemotherapy. In centres treating and monitoring haematological disorders, a high sensitivity for ‘abnormal lymphocytes’ is required. Thus, in these instances, laboratories should rely on the microscopic review as the gold standard.

The common reactive causes of monocytosis are chronic infections such as tuberculosis, inflammatory states, autoimmune disorders, and regenerating bone marrow post-insult.^18^ South Africa has the highest incidence of tuberculosis, with a prevalence of 852 cases (95% CI: 679–1026) per 100 000 population.^19^ Three (1.2%) samples flagged for ‘monocytosis’ by the analyser were determined to be FN. A microscopic examination of the PBS in these samples, however, revealed > 20% blasts in these samples. This is clinically relevant as these samples were from patients with malignancies, low WCCs, and recent chemotherapy exposure. Adding the ‘monocyte workflow optimisation’ rule set in the extended information processing unit of the analyser adds the function to distinguish reactive conditions from malignant monocytic conditions based on the presence of dysplasia and persistent (> 30 days) monocytosis (> 1.5 × 10^9^/L), and to recommend a blood smear examination in only malignant monocytic conditions.^18^ Furthermore, the combination of a monocyte count of > 1.5 × 10^9^/L with low haemoglobin (< 10 g/dL) or an abnormal or blast flag may warrant urgent PBS review to exclude a malignant aetiology. A microscopic review may be necessary in the clinical context in monocytosis cases with a low WCC, malignancy, and chemotherapy usage to exclude the presence of relapsed or residual disease.^18^

Overall, this study did not show a significant influence of the presence of HIV, previous or current coronavirus disease 2019 infection, or transplantation (solid organ or haematopoietic stem cell) on the sensitivity or specificity of the analyser, probably due to the low study numbers.

### Limitations

The study may be limited by the subjectivity of the primary investigator. This limitation was improved by adopting the assessment of a second observer. To the best of our knowledge, this is the first prospective study of this nature in the local population.

### Conclusion

The Sysmex XN-3000 automated FBC analyser generates valuable numerical (through the white cell differential and WPC channels) and qualitative (through internal protocol messages or flags) information that may assist laboratory staff in accurately assessing and interpreting PBS results and help pathologists and clinicians reach timeous and correct diagnoses.^15^ Our study confirmed that in laboratories focused on patients with haematological conditions, low WCC (< 1.5 × 10^9^/L) and undergoing chemotherapy or haematopoietic stem cell transplants, microscopy review remains the gold standard for the detection of blasts, abnormal lymphocytes, and monocytosis.
